# Robust Localization and Tracking of VRUs with Radar and Ultra-Wideband Sensors for Traffic Safety

**DOI:** 10.3390/s26051690

**Published:** 2026-03-07

**Authors:** Mouhamed Aghiad Raslan, Martin Schmidhammer, Ibrahim Rashdan, Fabian de Ponte Müller, Tobias Uhlich, Andreas Becker

**Affiliations:** 1 Faculty of Information Technology, Fachhochschule Dortmund—University of Applied Sciences and Arts, 44139 Dortmund, Germany; tobias.uhlich@fh-dortmund.de (T.U.); andreas.becker@fh-dortmund.de (A.B.); 2 German Aerospace Center, Institute of Communications and Navigation, (DLR), 82234 Wessling, Germany; martin.schmidhammer@dlr.de (M.S.); ibrahim.rashdan@dlr.de (I.R.); fabian.pontemueller@dlr.de (F.d.P.M.)

**Keywords:** traffic safety, radar, UWB, Vulnerable Road User (VRU), sensor fusion, Kalman filter

## Abstract

**Highlights:**

**What are the main findings?**

- The paper presents a novel approach to enhancing Vulnerable Road User (VRU) protection by integrating radar sensors and a widespread network of Ultra-Wideband (UWB) nodes through sensor fusion, in order to detect and track VRUs in urban environments.
- The experimental results demonstrate that the fusion of radar and UWB measurements reduces tracking uncertainties and improves the accuracy of VRU tracking, particularly in areas with blind spots or occlusions.

**What is the implication of the main finding?**

- The proposed system has the potential to contribute to the development of intelligent road infrastructures, enhancing urban traffic safety and reducing the risk of accidents involving VRUs, particularly in complex traffic scenarios where both vehicles and VRUs are present.
- The use of a widespread network of UWB nodes in conjunction with radar sensors enables the creation of a more comprehensive and robust VRU tracking system, which can be integrated into intelligent road infrastructures and contribute to the development of smarter and safer urban transportation systems, ultimately reducing the risk of accidents involving VRUs and improving overall traffic safety.

**Abstract:**

The increasing risk to Vulnerable Road Users (VRUs) at urban intersections necessitates advanced safety mechanisms capable of operating effectively under diverse conditions, including adverse weather like heavy rain. While optical sensors such as cameras and LiDAR often degrade in poor visibility, Radio Frequency (RF)-based systems offer resilient, all-weather tracking. This paper presents a novel approach to enhancing VRU protection by fusing two RF modalities: radar sensors and Ultra-Wideband (UWB) technology, a strong candidate for Joint Communication and Sensing (JCS). The research, conducted as part of the VIDETEC-2 project, addresses the limitations of existing vehicle-based and infrastructure-based systems, particularly in scenarios involving occlusions and blind spots. By leveraging radar’s environmental robustness alongside UWB’s precise, cost-effective short-range communication and localization, the proposed system delivers the framework for continuous vehicle and VRU tracking. The fusion of these sensor modalities, managed through a hybrid Kalman filter approach integrating an Unscented Kalman Filter (UKF) and an Extended Kalman Filter (EKF), allows reliable VRU tracking even in challenging urban scenarios. The experimental results demonstrate a reduction in tracking uncertainty and highlight the system’s potential to serve as a more accurate and responsive safety mechanism for VRUs at intersections. This work contributes to the development of intelligent road infrastructures, laying the foundation for future advancements in urban traffic safety.

## 1. Introduction

Vulnerable Road Users (VRUs), including pedestrians, cyclists, and motorcyclists, comprise a significant portion of traffic fatalities worldwide. In 2021, VRUs accounted for nearly half of all road fatalities within the European Union, underscoring the critical need for targeted interventions to increase their safety at intersections and other high-risk areas [1]. As urbanization and traffic density increase, the risk to VRUs, particularly at intersections, where vehicle paths frequently cross with those of pedestrians and cyclists, has become a pressing concern [2]. While essential, traditional road safety measures like traffic signals and road markings often fall short in complex scenarios, such as when vehicles perform right turning maneuvers or when VRUs enter a driver’s blind spot.

While Vehicle-to-VRU (V2VRU) communication can provide 360° awareness, it requires VRUs to carry active communication devices [3,4]. To avoid this dependency, modern vehicles are increasingly equipped with advanced onboard perception systems utilizing LiDAR, cameras, and radar. However, vehicle-centric systems are fundamentally limited by line-of-sight (LoS) occlusions as VRUs may be obscured by parked cars or buildings. Alternatively, infrastructure-based perception systems offer a more comprehensive approach to VRU protection by leveraging fixed sensors positioned at intersections and other high-risk areas [5]. These systems typically employ cameras mounted on infrastructure, providing a broader view of the environment from multiple angles. The data collected is processed to detect VRUs and potential hazards, with critical information transmitted to nearby vehicles via Cooperative Intelligent Transport Systems (C-ITSs) in the form of Cooperative Perception Messages (CPMs). While infrastructure-side cameras can mitigate the limitations of vehicle-based systems, they face challenges such as sensitivity to lighting and weather conditions, as well as privacy concerns. The need to comply with the General Data Protection Regulation (GDPR) and other privacy regulations often necessitates the anonymization of video streams and other data, which can complicate the implementation and operation of these systems.

To overcome both optical and privacy limitations, recent work has highlighted the role of Radio Frequency (RF)-based roadside sensing systems for VRU protection [6], including calibration, fusion strategies, synchronization, and trajectory-level safety assessment at intersections. RF technologies offer distinct advantages in environmental robustness and inherent data privacy. Radar sensors, for instance, are immune to poor visibility and adverse weather [7,8]. VIDETEC-2 project took the initiative in the (RF)-based VRU protection application [9] by installing static radars at fixed infrastructure positions, where it enables continuous VRU monitoring even when obstacles occlude the vehicle’s view [5]. Furthermore, radar’s ability to capture detailed kinematic features enables advanced VRU tracking and classification using machine learning [10,11,12,13]. To maximize coverage, roadside radars are typically installed at elevated positions, which inherently create near-field blind spots directly beneath the mounting location.

In parallel, Ultra-Wideband (UWB) technology, defined by IEEE 802.15.4, has emerged as a powerful tool to improve robustness in occluded or non-line-of-sight situations [14,15]. By transmitting ultra-short pulses over a wide frequency band, UWB achieves sub-decimeter accuracy for time-of-flight ranging and provides a detailed environmental fingerprint [16,17,18]. For sensing applications, UWB can be deployed as a multi-static radar network to detect moving reflections [19], or device-free localization (DFL) and multipath-enhanced DFL (MDFL) can be utilized to detect intersecting road users via link attenuation [17,20,21]. Given the previously demonstrated sub-meter indoor user localization [22], it is evident that UWB’s dual capability for communication and precise sensing makes it an ideal candidate for Joint Communication and Sensing (JCS). Note, however, that relying solely on UWB for environmental sensing necessitates a densely deployed network of nodes to maintain reliable localization accuracy across the observation area.

Building on the complementary strengths of radar and UWB, this paper presents a novel RF-based sensor fusion framework designed for the continuous tracking of both vehicles and VRUs at urban intersections, also overcoming the individual sensor specific limitations. The proposed system leverages a hybrid Kalman filter architecture, combining an Extended Kalman Filter (EKF) to process radar detections with an Unscented Kalman Filter (UKF) to integrate information from power changes measured in a widely distributed UWB network. Fusing these modalities provides redundant measurements that help reduce positional uncertainty and compensate for near-field radar blind spots, supporting continuous trajectory estimation in occluded traffic scenarios.

In order to evaluate the localization and tracking performance of the proposed radar–UWB fusion algorithm, the sensing results are compared against ground-truth trajectories from an RTK-GNSS high-precision localization system. The experimental evaluation focuses on a single GNSS-equipped VRU per run. Restricting each trial to a single target isolates the behavior of the fusion pipeline, particularly its handling of blind spots, and provides a clear error metric without the additional complexity of multi-target data association. Please note that although the evaluation considers a single trajectory in isolation to ensure precise error measurement, the implemented fusion architecture inherently supports the simultaneous detection and tracking of multiple road users.

The remainder of this paper is organized as follows. Section 2 details the sensor setup, data acquisition, and the proposed hybrid Kalman filter fusion methodology. Section 3 describes the experimental setup and the practical challenges encountered during field testing. The results are presented in Section 4, and the performance of the tracking system is evaluated, demonstrating its viability as a reliable safety mechanism for complex traffic scenarios. Finally, Section 5 concludes.

## 2. RF-Based Vehicle and VRU Tracking System

**Figure 1:**
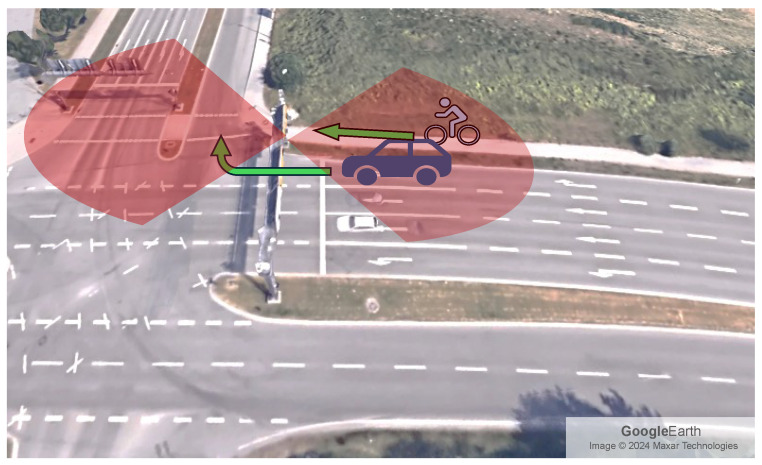
A potentially dangerous scenario of a cyclist and a right-turning vehicle, both having right of way to proceed into the intersection. The red areas show the field of view of the 2 radars installed on the gantry. Source: Google Earth.

This work addresses the detection and safety of VRUs at signalized intersections, specifically focusing on scenarios where right-turning vehicles and crossing VRUs share a concurrent green-signal phase. Due to the lateral offset of the pedestrian/cyclist lane from the main carriageway, the driver’s side-mirror field of view is often occluded (see Figure 1). To mitigate the occluded field of view, we propose a multimodal sensor fusion framework that integrates detections from infrastructure-based radar units with measurements from a distributed network of UWB nodes. These fused data streams are processed by a joint-state tracking algorithm that simultaneously estimates the trajectories of both vehicles and VRUs. When the tracker identifies a predicted trajectory intersection within a predefined safety margin, a targeted warning can be issued to the vehicle to prevent imminent conflict. In the following, we first describe the processing of the individual sensors, i.e., radar and UWB, and then we outline the sensor-fusion algorithm used for vehicle and VRU tracking.

### 2.1. Radar Processing

Measurements obtained from the radar undergo several pre-processing steps before entering the tracking algorithm. In this subsection, the steps of achieving the final shape and features of the radar measurements are explained.

#### 2.1.1. Radar’s Signal Model and Detections

The Frequency Modulated Continuous Wave (FMCW) radar modules from IMST GmbH operate in the 77/79 GHz frequency bands [23], leveraging sophisticated signal processing to detect and track objects with high precision.

The transmitted chirp signal $x_{T(t)}$ is generated through frequency modulation and is represented as follows:

(1) $$x_{T}\left(t\right)=A_{Tx}\cos\left(2\pi f_{0}t+\frac{\pi Bt^{2}}{T_{c}}\right),$$

where $A_{Tx}$ denotes the real-valued amplitude of the transmitted signal, $f_{0}$ denotes the starting frequency, *B* is the bandwidth, and $T_{c}$ represents the duration of the chirp. The received signal from a target reflection $x_{R}\left(t\right)$ is a time-delayed and phase-shifted version of the transmitted signal:

(2) $$x_{R}\left(t\right)=A_{Rx}\cos\left(2\pi f_{0}\left(t-\tau \right)+\frac{\pi B{\left(t-\tau \right)}^{2}}{T_{c}}\right),$$

where $A_{Rx}$ denotes the real-valued amplitude of the received signal, τ is the round-trip delay. Mixing the received and transmitted signals yields the intermediate frequency signal $x_{IF}$:

(3) $$x_{IF}\approx \frac{1}{2}\left[\cos\left(\phi_{T}\left(t-\tau \right)-\phi_{T}\left(t\right)\right)+\cos\left(\phi_{T}\left(t-\tau \right)+\phi_{T}\left(t\right)\right)\right],$$

where $\phi_{T}\left(t\right)$ represents the phase of the transmitted signal. The difference in frequency between the transmitted and the received signals is the beat frequency $f_{b}$. It serves as a vital factor in computing the range *d* of the target, and it can be mathematically expressed as

(4) $$f_{b}=\frac{B\tau }{T_{c}}=\frac{2Sd}{c},$$

where *S* is the slope of the frequency change over the chirp duration and *c* is the speed of light. We can obtain the beat frequency by performing Fast Fourier Transform (FFT) on the $x_{IF}$ signal. Hence, the range *d* to the target can then be calculated:

(5) $$d=\frac{f_{b}c}{2S}$$

In order to obtain the velocity measurement, the phase shift Δϕ generated by the small displacement of the moving target should be calculated using

(6) $$\Delta \phi =2\pi f_{0}\Delta \tau =\frac{4\pi \Delta d}{\lambda },$$

where Δd represents the change in the target’s position, and λ denotes the wavelength of the signal.

Assuming the target is moving with a radial speed *v* within the chirp duration $T_{c}$, the change in its position Δd can be expressed as

(7) $$\Delta d=vT_{c}$$

Substituting in Equation (6) yields

(8) $$v=\frac{\lambda \Delta \phi }{4\pi T_{c}}$$

The maximum measurable velocity is determined by satisfying a condition, which states that the phase shift Δϕ remains within a certain range, $|\Delta \phi |<180^{\circ }$, for unambiguous velocity measurement. Thus, the maximum measurable velocity $v_{max}$ can be calculated as

(9) $$v_{\max}=\frac{\lambda }{4T_{c}}$$

According to the manufacturer’s manual [24], the radar module’s firmware performs a series of signal processing steps. Starting with the Analog/Digital Converter (ADC), the signals from the radar’s $R_{x}$ section are sampled, digitized and stored in the processor’s memory. Then Range and Doppler FFTs are applied along the time domain and the chirp sequence respectively, then it the data from all receiver channels are combined for noise suppression to create a Range-Doppler Map (RDM). Potential targets are identified Constant False Alarm Rate (CFAR), with additional parameters like magnitude, elevation, and azimuth angles calculated.

During the experiment, the radar was configured for continuous measurement with an interval of 60 ms, allowing for real-time data acquisition and processing. The radar processing mode was set to obtain detections, with a range resolution of 0.16 m and a velocity resolution of 0.665 m/s.

#### 2.1.2. Linearization

In radar signal processing, linearization is a crucial step for transforming the raw radar detections from their native polar coordinate system (range, Doppler-velocity, azimuth, and elevation) into a Cartesian coordinate system. This transformation facilitates the application of tracking algorithms, such as the Kalman filter, which operate more effectively in a linear, Cartesian space [25]. The necessity of linearization arises from the nature of the Kalman filter, which assumes linear state variable models [26]. The Kalman filter predicts the future state of an object based on its current state and updates this prediction using new measurements. In a Cartesian coordinate system, the motion dynamics of an object can be described using linear equations. Conversely, the measurements in polar coordinate system introduce nonlinearities that complicate the tracking process [27].

##### Nonlinear Measurement Model

The radar system provides measurements in terms of range (*r*), azimuth (ϕ), elevation (θ), and range rate ($\dot{r}$). The nonlinear measurement model can be expressed as

(10) $$z_{\mathrm{radar}}=\left[\begin{array}{c} r\\ \phi \\ \theta \\ \dot{r} \end{array}\right]=h_{\mathrm{radar}}\left(x\right)+v_{\mathrm{radar}},$$

where $z_{\mathrm{radar}}$ is the measurement vector, $h_{\mathrm{radar}}\left(x\right)$ is the nonlinear function relating the state vector x to the measurements and $v_{\mathrm{radar}}$ is the measurement noise vector, assumed to be Gaussian with zero mean and covariance matrix $R_{\mathrm{radar}}$. The state vector x typically includes the position and velocity components in Cartesian coordinates:

(11) $$x={\left[\begin{array}{cccccc} x, & \dot{x}, & y, & \dot{y}, & z, & \dot{z} \end{array}\right]}^{T}$$

The nonlinear measurement function $h_{\mathrm{radar}}\left(x\right)$ is defined as

(12) $$h_{\mathrm{radar}}\left(x\right)=\left[\begin{array}{c} \sqrt{x^{2}+y^{2}+z^{2}}\\ \arctan\left(\frac{y}{x}\right)\\ \arccos\left(\frac{z}{\sqrt{x^{2}+y^{2}+z^{2}}}\right)\\ \frac{x\dot{x}+y\dot{y}+z\dot{z}}{\sqrt{x^{2}+y^{2}+z^{2}}} \end{array}\right]$$

$v_{\mathrm{radar}}$ is Gaussian distributed with measurement noise covariance $R_{\mathrm{radar}}$

(13) $$v_{\mathrm{radar}}∼N\left(0,R_{\mathrm{radar}}\right)$$

The measurement noise covariance matrix $R_{\mathrm{radar}}$ is given by

(14) $$R_{\mathrm{radar}}=\left[\begin{array}{cccc} \sigma_{r}^{2} & 0 & 0 & 0\\ 0 & \sigma_{\phi }^{2} & 0 & 0\\ 0 & 0 & \sigma_{\theta }^{2} & 0\\ 0 & 0 & 0 & \sigma_{\dot{r}}^{2} \end{array}\right],$$

where $\sigma_{\phi }^{2}$ is the variance of the azimuth measurement noise, $\sigma_{\theta }^{2}$ is the variance of the elevation measurement noise, $\sigma_{r}^{2}$ is the variance of the range measurement noise and $\sigma_{\dot{r}}^{2}$ is the variance of the range rate measurement noise.

##### Linearization of the Measurement Model

To apply the Extended Kalman Filter (EKF), we need to linearize the nonlinear measurement function $h_{\mathrm{radar}}\left(x\right)$ around the current state estimate $x_{k}$. This is done by computing the Jacobian matrix H of $h_{\mathrm{radar}}\left(x\right)$ with respect to the state vector x [25].

The Jacobian matrix H is defined as

(15) $$H={\left.\frac{\partial h_{\mathrm{radar}}\left(x\right)}{\partial x}\right|}_{x=x_{k}}$$

where $x_{k}$ is the current state estimate at the time instance *k* [28].

Given the measurement function in (12), we compute the partial derivatives of each component of $h_{\mathrm{radar}}\left(x\right)$ with respect to each state variable in (11) [29].

The linearized measurement model is then

(16) z≈Hx+v.

##### Linearizing Position and Velocity Components

The process of linearizing radar data into position components involves converting polar coordinates (r,θ,ϕ) into Cartesian coordinates (x,y,z).

(17) $$\begin{array}{cc} x & =r\sin(\theta )\cos(\phi )\\ y & =r\sin(\theta )\sin(\phi )\\ z & =r\cos(\theta ) \end{array}$$

In a general scenario, linearizing the velocity components $\left(\dot{x},\dot{y},\dot{z}\right)$ involves complex calculations due to the dependency on the derivatives of the radar measurements. However, for our application, we utilize the knowledge of the environment to simplify the problem. The locations of the static radars, the path of the vehicles, and the targets’ heading are known and relatively constant. Utilizing the knowledge of the environment, it is possible to estimate the true velocity of the target. Considering the targets are moving in a straight line on the x-axis towards the radar, as in the scene shown in Figure 1, the estimated target’s true velocity $v_{x}$ can be calculated directly from the range rate $\dot{r}$ and the radar measurements (θ,ϕ) [30,31] as follows:

(18) $$v_{x}=\frac{\dot{r}}{\cos(\phi )\cos(\theta )}$$

Please note that highly tangential motion relative to the radar line-of-sight, abrupt stop-go, or strong turning can affect the accuracy of the Doppler-to-Cartesian velocity mapping.

#### 2.1.3. Clustering

In this application, the Density-Based Spatial Clustering of Applications with Noise (DBSCAN) algorithm was applied to radar data to cluster several radar detections reflected from a single target. The DBSCAN algorithm operates based on two primary parameters: the radius ϵ and the minimum number of points minPts. The algorithm categorizes points into core points, border points, and noise based on these parameters. For a point *p*:

1. Epsilon neighborhood: the set of points within a distance ϵ from *p*:
  (19) $$N_{\epsilon }\left(p\right)=\left\{q\in D|d\left(p,q\right)\leq \epsilon \right\}$$
  where *D* is the dataset and d(p,q) is the distance between points *p* and *q*.
2. Core point: a point *p* is a core point if
  (20) $$|N_{\epsilon }\left(p\right)|\geq \mathrm{minPts}$$

Based on these definitions, DBSCAN identifies clusters as maximal sets of density-connected points and labels points that do not belong to any cluster as noise [32,33]. However, targets whose Radar Cross-Section (RCS) is only enough to reflect a single detection can be considered as noise or outliers by DBSCAN. Hence, such outliers (possible detections) are still considered as an input into the tracking algorithm by setting the minPts=1. The tracks initiator/deleter, in return, handles such detections based on the results from the data association algorithm in case they are clutter.

Prior to applying the clustering algorithm, normalizing the data is essential due to different axes the data is represented on, i.e., position axes and velocity axes, to ensure that all features contribute equally to the distance calculations. This step is crucial because DBSCAN relies on distance measures to define neighborhoods and identify core points, and features with different scales can bias these calculations. We employed Min-Max scaling for normalization, which transforms each feature value *s* to a value $s^{\prime }$ within the range [0, 1] using the formula

(21) $$s^{\prime }=\frac{s-\min(s)}{\max(s)-\min(s)}$$

where min(s) and max(s) are the minimum and maximum values of the feature, respectively. Normalizing the data helps mitigate the dominance of features with larger scales and facilitates the selection of appropriate ϵ values for the DBSCAN algorithm, which is set to ϵ=0.05 after normalization [34,35].

### 2.2. Ultra Wideband Measurements

Complementing the radar system described above, we consider a network of UWB nodes supporting the localization of VRUs. The transceiving UWB nodes are spatially distributed along the pedestrian/cyclist path, and the locations are precisely measured in advance. For each network link *l* of the UWB system, i.e., the link between transmitting node ${\mathrm{Tx}}_{i}$ and a receiving node ${\mathrm{Rx}}_{j}$, we observe the received power ${\hat{\gamma }}_{l}\left(t\right)$. Since we want to obtain information about the target’s location from the induced fading, we need to observe the changes in the received power in particular. To establish a reference power level, we therefore collect received power data during an initialization period and calculate the average, i.e., ${\bar{\gamma }}_{l}$, as detailed in [21]. By subtracting the reference power level from the measured power, we can define the power changes in logarithmic domain as

(22) $$z_{\mathrm{UWB}}={\left[\ldots ,{\hat{\gamma }}_{l}\left(t\right)-{\bar{\gamma }}_{l},\ldots \right]}^{T}$$

for all considered network links l∈{1,…,L}.

#### 2.2.1. Measurement Model

Given the measurement vector of the UWB system in (22), we can define the measurement model as

(23) $$z_{\mathrm{UWB}}=h_{\mathrm{UWB}}\left(x\right)+v_{\mathrm{UWB}}\;,$$

where $h_{\mathrm{UWB}}\left(x\right)$ is the nonlinear function relating the state vector x to the measurements and $v_{\mathrm{UWB}}$ is the measurement noise vector of the UWB system, assumed to be Gaussian with zero mean and covariance matrix

(24) $$R_{\mathrm{UWB}}=\mathrm{diag}\left(\ldots ,\sigma_{\gamma_{l}}^{2},\ldots \right),\;\mathrm{for}\;l\in \left\{1,\ldots ,L\right\},$$

where $\sigma_{\gamma_{l}}^{2}$ is the variance of the *l*-th network link.

The nonlinear measurement function for an individual network link *l* is defined as

(25) $${\left[h_{\mathrm{UWB}}\left(x\right)\right]}_{l}=\phi_{l}e^{\xi_{l}\left(x\right)/\kappa_{l}}\;,$$

with $\phi_{l}$ as the maximum modeled power change in dB and $\kappa_{l}$ as the spatial decay rate. The state-dependent excess path length $\xi_{l}\left(x\right)$ is defined as

(26) $$\xi_{l}\left(x\right)=∥r_{l}^{\mathrm{Tx}}-r∥+∥r_{l}^{\mathrm{Rx}}-r∥-∥r_{l}^{\mathrm{Tx}}-r_{l}^{\mathrm{Rx}}∥\;,$$

where $r_{l}^{\mathrm{Rx}}$ and $r_{l}^{\mathrm{Tx}}$ refer to the known positions of the receiving and transmitting node, respectively, and $r={\left[x,y,z\right]}^{⊺}$ refers to the target position being part of the state vector x.

#### 2.2.2. Link Selection

The purpose of the UWB network is to complement the radar system by providing additional location information from target induced fading measurements. As the network is widely distributed along the bicycle lane, it is essential to preselect the most relevant network links for the update process, improving the computational efficiency and the accuracy of the tracking system.

The pre-selection process is guided by the proximity of the network links to the target detections provided by the radar system or the tracking system, respectively. This proximity is quantified by the excess path length of the current target state $x_{k}$ at time instance *k*. A link is selected for update if the excess path length is less than or equal to a threshold $\xi_{\mathrm{th}}$. That means, using (26), the selection can be expressed by the boolean operation

(27) $$s_{k,l}=\left(\xi_{l}\left(x_{k}\right)\leq \xi_{\mathrm{th}}\right),$$

where $s_{l}$ is set to 1 if the condition $\xi_{l}\left(x_{k}\right)\leq \xi_{\mathrm{th}}$ is true, which indicates that the target is expected to be in the proximity of the link, and 0 otherwise. Accounting for the physical dimensions of the considered targets, i.e., pedestrians and cyclists, in this work, we set the threshold to $\xi_{\mathrm{th}}=1m$. Finally, the overall selection vector $s_{k}$ is

(28) $$s_{k}={\left[\ldots ,s_{k,l},\ldots \right]}^{T},\;\mathrm{for}\;l\in \left\{1,\ldots ,L\right\}.$$

In order to select the relevant network links, the selection vector is applied to the measurement vector in (22) by

(29) $$z_{k}^{\mathrm{sel}}=s_{k}\circ z_{\mathrm{UWB}},$$

where ∘ denotes the element-wise product. Similarly, we can select the relevant elements of the measurement noise covariance matrix in (24) as

(30) $$R_{k}^{\mathrm{sel}}=S_{k}R_{\mathrm{UWB}}S_{k},$$

where $S_{k}=\mathrm{diag}\left(s_{k}\right)$ is the diagonal selection matrix constructed from the selection vector $s_{k}$ of (28).

### 2.3. Sensor Fusion

**Figure 2:**
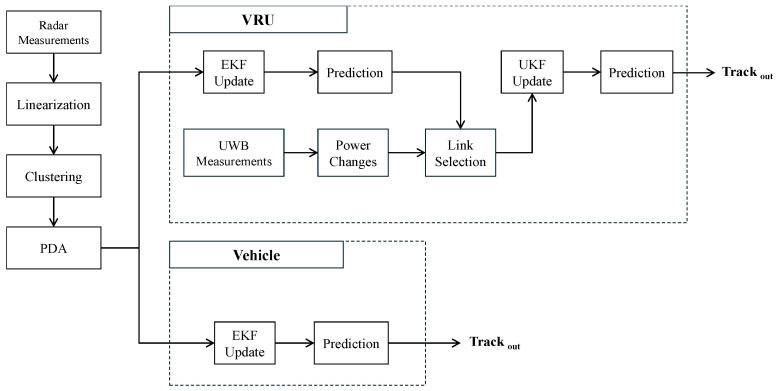
A block diagram of the sensor fusion process utilizing EKF and UKF updaters based on target’s class.

After processing the measurements from the radar sensors, the class of the target, based on the single-frame target classification model developed in [12], triggers the tracking algorithm. A Kalman-Filter-based tracking algorithm is utilized to fuse the measurements obtained from radar and UWB sensors. However, due to the difference in measurement models between the two types of sensors, two types of updaters are integrated in this algorithm, an EKF updater and a UKF updater. The EKF is always in operation; however, the UKF updater is triggered only upon the availability of the UWB measurements as illustrated in Figure 2. The main tracking algorithm is a multi-target tracking system designed to accurately estimate the trajectories of multiple objects over time. The primary objective of this tracking algorithm is to manage the association between measurements (detections) and tracked objects (tracks), predict future states, and update the tracks based on new measurements. The EKF component follows standard radar tracking practice (prediction and update on radar detections). The contribution of this work is the fusion architecture that augments radar tracking with UWB measurements through an additional UKF update when UWB measurements are available, enabling continuous VRU tracking and reduced uncertainty during radar blind-spot and outage intervals.

In this application, the UWB network nodes are distributed along the sides of the VRU lane to measure the attenuations induced by a passing VRU. Since the network is focused on measuring the position of a passing VRU, the UKF updater associated with the measurements obtained from the network is triggered only when a VRU is present in the scene.

#### 2.3.1. Extended Kalman Filter (EKF)

The tracking algorithm begins with the processed measurements, which are in a 6-dimensional state vector format as in (11). These detections are then processed by the Probabilistic Data Association (PDA) hypothesizer which generates multiple hypotheses regarding the association between detections and existing tracks.

For a given track and *N* detections, the probability $\beta_{i}\left(k\right)$ that detection *i* is associated with the track at time *k* is given by

(31) $$\beta_{i}\left(k\right)=\{\begin{array}{cc} \frac{L_{i}\left(k\right)}{1-P_{D}P_{G}+\sum_{j=1}^{m(k)}L_{j}\left(k\right)}, & i=1,\ldots ,m\left(k\right)\\ \frac{1-P_{D}P_{G}}{1-P_{D}P_{G}+\sum_{j=1}^{m(k)}L_{j}\left(k\right)}, & i=0 \end{array}$$

where $P_{D}$ is the detection probability, $P_{G}$ is the gate probability, m(k) is the number of detections at time *k* and $L_{i}\left(k\right)$ is the likelihood of the *i*-th detection at time *k*. The likelihood $L_{i}\left(k\right)$ is calculated by

(32) $$L_{i}\left(k\right)=\frac{N\left[z_{i}\left(k\right);\hat{z}\left(k|k-1\right),S\left(k\right)\right]P_{D}}{\lambda }$$

where λ is the clutter density, $N[z_{i}\left(k\right);\hat{z}\left(k|k-1\right),S\left(k\right)]$ is the likelihood ratio of the measurement $z_{i}\left(k\right)$ originating from the track target rather than clutter, $\hat{z}\left(k|k-1\right)$ is the predicted measurement, and S(k) is the innovation covariance matrix [36]. This formulation ensures that the association probabilities are computed exclusively, maintaining the integrity of the tracking process by accounting for both actual detections and missed detection scenarios.

The EKF predictor uses these hypotheses to predict the future states of the tracks, where it utilizes a constant velocity model to predict the future state of each track, which can be described as

(33) $$x_{k|k-1}=F_{k}x_{k-1}+w_{k}$$

where

(34) $$F_{k}=\left[\begin{array}{cccccc} 1 & \Delta t & 0 & 0 & 0 & 0\\ 0 & 1 & 0 & 0 & 0 & 0\\ 0 & 0 & 1 & \Delta t & 0 & 0\\ 0 & 0 & 0 & 1 & 0 & 0\\ 0 & 0 & 0 & 0 & 1 & \Delta t\\ 0 & 0 & 0 & 0 & 0 & 1 \end{array}\right]$$

and $w_{k}$ is the process noise assumed to be Gaussian with zero mean and covariance Q [37].

The Joint Probabilistic Data Association (JPDA) calculates the joint probabilities of all possible associations between tracks and measurements. For each track *i* and measurement *j*, the association probability is

(35) $$\beta_{ij}=\frac{P\left(H_{ij}\right)L\left(Z_{ij}\right)}{\sum_{k}P\left(H_{ik}\right)L\left(Z_{ik}\right)}$$

where $P(H_{ij})$ is the prior probability of hypothesis $H_{ij}$, and $L(Z_{ij})$ is the likelihood of the measurement $Z_{ij}$ given the hypothesis. The association probabilities are used to update the weights of the hypotheses [38]. Once associations are determined, the EKF updater refines the state estimates of the tracks based on the new measurements. The EKF updater uses the linear Gaussian measurement model, as in (16), to update the predicted state with new measurements. The update process is

(36) $$K_{k}=P_{k|k-1}H_{k}^{T}{\left(H_{k}P_{k|k-1}H_{k}^{T}+R_{\mathrm{linear}k}\right)}^{-1}$$

(37) $$x_{k}=x_{k|k-1}+K_{k}\left(z_{k}-H_{k}x_{k|k-1}\right)$$

(38) $$P_{k}=P_{k|k-1}-K_{k}H_{k}P_{k|k-1}$$

where $K_{k}$ is the Kalman gain, $P_{k}$ is the error covariance matrix, and $R_{\mathrm{linear}k}$ is the measurement noise covariance matrix [29].

#### 2.3.2. Unscented Kalman Filter (UKF) Update

After performing the EKF prediction step, as in Figure 2, using the constant velocity model described in (33), the predicted state $x_{k|k-1}^{\mathrm{EKF}}$ and the associated covariance matrix $P_{k|k-1}^{\mathrm{EKF}}$ serve as the prior for the UKF update. The UKF is utilized to incorporate the measurements of changes in the received power of the UWB system.

For the UKF update, we first generate a set of sigma points $\chi_{i}$ that capture the uncertainty in the state estimate. These sigma points are deterministic samples chosen to represent the mean and covariance of the state distribution. The sigma points are computed as follows

(39) $$\chi_{i}=x_{k|k-1}^{\mathrm{EKF}}\pm \sqrt{\left(n+\lambda \right)P_{k|k-1}^{\mathrm{EKF}}}$$

where *n* is the dimension of the state vector, and λ is a scaling parameter defined by $\lambda =\alpha^{2}\left(n+\kappa \right)-n$, with α and κ as tuning parameters that control the spread of the sigma points [39]. For the experimental evaluation presented in Section 4, the tuning parameters were set to standard literature values of α=0.1 to minimize non-local sampling effects, and κ=1 to guarantee positive-definite covariance matrices [39]. Each sigma point is then propagated through the nonlinear measurement model defined in (25) as

(40) $$z_{i,k}^{\sigma }=s_{k}\circ h_{\mathrm{UWB}}\left(\chi_{i}\right),$$

considering the relevant network links according to the link selection vector $s_{k}$ defined in (28). This yields a set of transformed sigma points in the measurement space. The predicted measurement mean ${\hat{z}}_{k}$ and the innovation covariance $P_{zz}$ are then computed as the weighted average of these transformed sigma points as

(41) $${\hat{z}}_{k}=\sum_{i=0}^{2n}W_{i}^{m}z_{i,k}^{\sigma },$$

and

(42) $$P_{zz}=\sum_{i=0}^{2n}W_{i}^{c}\left(z_{i,k}^{\sigma }-{\hat{z}}_{k}\right){\left(z_{i,k}^{\sigma }-{\hat{z}}_{k}\right)}^{T}+R_{k}^{\mathrm{sel}},$$

where $W_{i}^{m}$ and $W_{i}^{c}$ are the weights associated with the mean and covariance calculations, and $R_{k}^{\mathrm{sel}}$ is the measurement noise covariance of (30) according to the link selection.

The Kalman gain is calculated as

(43) $$K_{k}=P_{xz}P_{zz}^{-1},$$

where $P_{xz}$ denotes the cross-covariance matrix between state and measurement, which is defined as

(44) $$P_{xz}=\sum_{i=0}^{2n}W_{i}^{c}\left(\chi_{i}-x_{k|k-1}^{\mathrm{EKF}}\right){\left(z_{i,k}^{\sigma }-{\hat{z}}_{k}\right)}^{T}\;.$$

Finally, the state estimate and the covariance matrix are updated using the Kalman gain and the power change measurements $z_{k}^{\mathrm{sel}}$ of (29) as

(45) $$x_{k|k}^{\mathrm{UKF}}=x_{k|k-1}^{\mathrm{EKF}}+K_{k}\left(z_{k}^{\mathrm{sel}}-{\hat{z}}_{k}\right)$$

and

(46) $$P_{k|k}^{\mathrm{UKF}}=P_{k|k-1}^{\mathrm{EKF}}-K_{k}P_{zz}K_{k}^{T}\;.$$

The updated state and covariance reflect the integration of the nonlinear power change measurements of the UWB system. As shown in Figure 2, the process now proceeds with the prediction step defined in (33), using the updated state and covariance as input, maintaining the cyclic nature of the hybrid tracking system.

## 3. Measurement Setup

**Figure 3:**
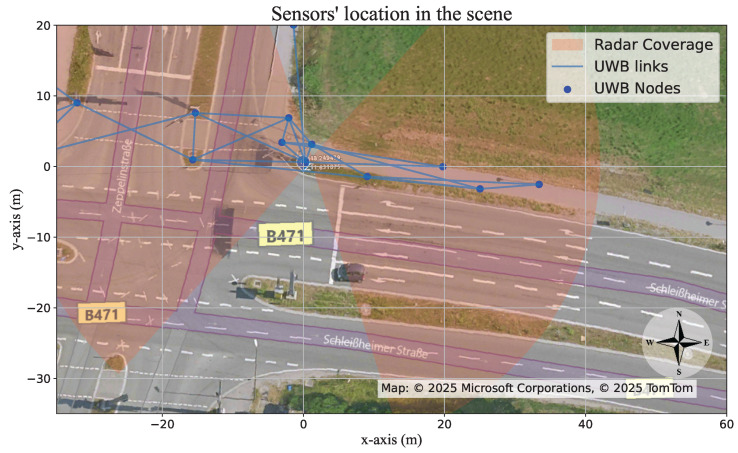
A satellite image of the scene with the location of the UWB nodes and the field of view of the two radars. Source: Bing Maps.

Two static radar sensors are installed on a gantry at an intersection. The gantry is located on one of the incoming roads to the intersection, on which the two radar sensors are installed on each side; one is facing east (the pedestrian/cyclist path), and the other is facing west (the intersection). The radar facing east monitors the trajectories of VRUs and vehicles that approach the right-turning lane. Conversely, the radar facing west observes VRUs that arrive from the opposite direction, i.e., VRUs approaching the intersection from the west, as well as vehicles that travel westward, from the east, in the same right-turning lane. To maximize the observable range for both VRUs and vehicles, each radar was yaw-adjusted so that the main lobe of its antenna pattern points toward the farthest detectable point of an approaching target. This configuration inevitably creates a blind-spot directly beneath each sensor. The respective areas of radar coverage are highlighted in red in Figure 1 and Figure 3. Typically, mutual interference from approaching vehicles equipped with active radar sensors may occasionally cause missed detections or increased clutter. In our observations, however, these effects were typically brief, likely because the gantry-mounted radar operated at a higher elevation than the vehicle-mounted sensors and because the manufacturer’s optimized CFAR algorithm reduced clutter in such situations. In addition to the two radar sensors, 13 UWB nodes are installed alongside the pedestrian lane, as indicated by the blue dots in Figure 3.

The height of the gantry is 8.5 meters. Since the radars are installed on top of the gantry, a significant blind spot below the radars is created, which leads to a loss of radar detections and, hence, to an increasing uncertainty in the VRU’s location. To avoid this, detections from the UWB nodes are incorporated into the tracker while the VRU is in the radars’ blind spot area.

The UWB sensing network comprises 13 fixed nodes that are mounted at different heights, ranging from 0.1 m to approximately 1.5 m, and positioned at various points around the intersection. The locations of the UWB nodes are depicted in Figure 3. Each UWB node consists of a Qorvo DWM1000 UWB transceiver [40] and a Raspberry Pi computer. The DWM1000 complies with the IEEE 802.15.4-2011 UWB standard. [41]

The Raspberry Pi runs driver software that controls the transceiver, generates and schedules the transmission of sensing messages according to a Time-Division Multiple Access (TDMA) scheme, and stores the TX and RX timestamps for every received message. In addition to the timestamps, the DWM1000 stores the Channel Impulse Response (CIR) as complex 16-bit raw samples in an internal accumulator memory that can be accessed by the Raspberry Pi. Each UWB node was tuned to a frequency of 3.9936 GHz and was broadcasting a sensing message every 120 ms using 500 MHz bandwidth. The TX power was set to −8 dBm/MHz. For further technical information on UWB nodes, the TDMA scheme, timing and ranging, please refer to [42]. The individual parameters required for the measurement model in (25) are retrieved empirically. The values for maximum modeled power changes, $\phi_{l}$, range from −8 dB to −0.72 dB, and the values for the decay rate, $\kappa_{l}$, from 0.01 m to 0.50 m. The elements of the covariance matrix in (24) take values between 0.45 dB and 1.88 dB.

**Figure 4:**
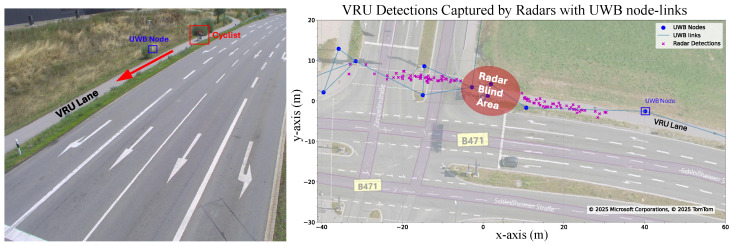
A cyclist on the VRU lane crossing the intersection and the radar detections corresponding to the cyclist. Source: Bing Maps.

The aim of this experiment was to detect, locate and track a VRU in a real-life traffic situation using the sensor setup described above. The left image in Figure 4 shows an example of a cyclist riding toward the intersection in the VRU lane, while the right image displays the corresponding radar detections in magenta. Please note the blind spot beneath the radars, highlighted in red in Figure 4, appearing as a conspicuous gap in the magenta points. In total, we considered seven runs in this experiment: three runs with a cyclist, two runs with an e-scooter, and two runs with a pedestrian.

In order to evaluate the proposed EKF/UKF radar–UWB sensor fusion to track the VRU, an independent ground truth system is required. Therefore, the VRU was equipped with an RTK-based GNSS system that records its position at a rate of 10 Hz during each run. In addition, these experiments were performed at Providentia++ test field, which is a camera-based high-accuracy traffic detection and tracking system, to ensure an advantage of the test field’s camera-based ground truth system [43].

During the experimental runs a number of ground-truth samples were lost or corrupted. Missing points originate from temporary occlusions of the VRU in the camera-based system and from temporary loss of the RTK-GNSS fix. To maintain continuous reference tracks for the evaluation, missing segments were reconstructed via linear interpolation between the nearest valid, high-accuracy RTK points [44,45]. To ensure the integrity of the evaluation data, we quantified the spatial and temporal gaps introduced by these outages. Histogram analysis of the tracking data revealed that interpolation events were infrequent, with the absolute worst-case spatial gap measuring only 1.2 m. Given the physical dimensions of a typical VRU and their constrained kinematic mobility profiles, i.e., low velocities and bounded accelerations, a maximum interpolation distance of 1.2 m falls well within the spatial footprint and predictable movement bounds of the target. Consequently, linear interpolation over these minor gaps introduces negligible error and is strongly justified.

## 4. Results

**Figure 5:**
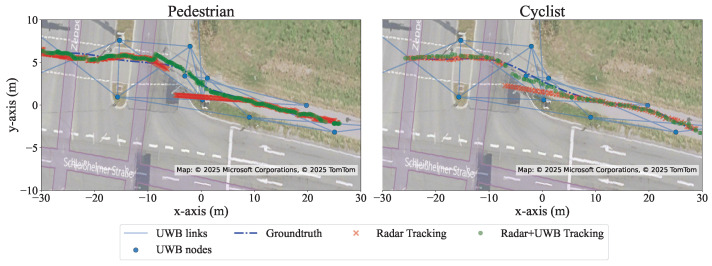
Radar-only tracks (in red) follow the predictions of the motion model only within that blind spot, where the tracks deviate from the actual path, whereas fusion tracks (in green) continue through the pedestrian/cyclist path towards the intersection as well as remaining close to the ground truth (in blue). Map Source: Microsoft Maps.

The experimental results were evaluated using multiple metrics to capture different performance aspects of the proposed method. This multi-metric approach mitigates reliance on the interpolated ground truth segments, ensuring a more robust and unbiased evaluation. Before presenting the quantitative analysis, Figure 5 provides a visual representation of two sample runs. The figure clearly reveals how the radar’s blind-spot degrades the raw tracks and how the fused solution restores continuity.

**Figure 6:**
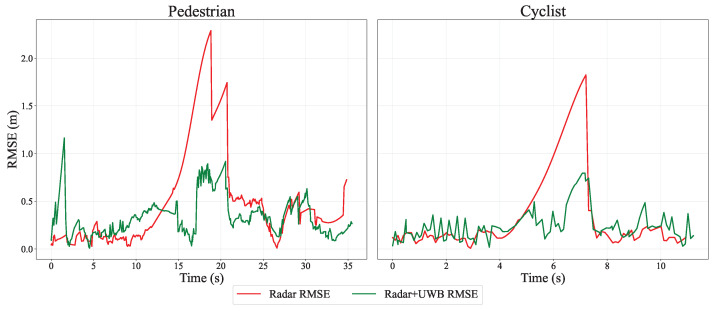
An RMSE metric over time of the two runs corresponding to Figure 5, one of a cyclist and one of a pedestrian, showing that RMSE values for both runs increase during the radars’ blind spot.

The primary metric used to evaluate the results is the root-mean-square error (RMSE), defined as

(47) $${\mathrm{RMSE}}_{k}=\sqrt{\frac{1}{N}\sum_{i=1}^{N}{\left(x_{k}^{i}-{\hat{x}}_{k}^{i}\right)}^{2}}$$

where *i* is the track number at the timestep *k*, and *N* is the total number of tracks at the timestep. The RMSE results for two runs are shown in Figure 6. In the radar-only configuration, both the pedestrian and cyclist runs exhibit a significant increase in RMSE in the radar blind spot. In contrast, the radar–UWB sensor fusion keeps the RMSE close to 0.5 m around the blind spot and prevents it from exceeding 1 m. At some time steps, particularly when the target is within radar coverage, the radar-only case is slightly more stable, whereas the fusion output exhibits jitter of about 0.15 m.

This jitter is a direct result of the asynchronous data acquisition between the radar, UWB sensors, and the ground-truth system. To address clock alignment, all incoming sensor measurements are timestamped upon arrival at the central processing unit using a shared system clock. Because the sensors operate at varying frequencies without hardware-level synchronization, the EKF/UKF handles the asynchronous updates by using the exact time difference Δt between consecutive measurement timestamps for the prediction step. For evaluation, each tracker output is temporally paired with the nearest GNSS sample. If the time difference exceeds a 50 ms threshold, which corresponds to half the sampling interval of the filter’s 10 Hz update rate, the sample is discarded. While spatial interpolation was utilized to reconstruct missing GNSS segments, no temporal resampling or artificial alignment was applied to the incoming sensor streams. Consequently, the natural end-to-end timing and minor processing latencies are preserved, which manifests as the observed 0.15 m jitter during higher-speed cyclist runs.

**Figure 7:**
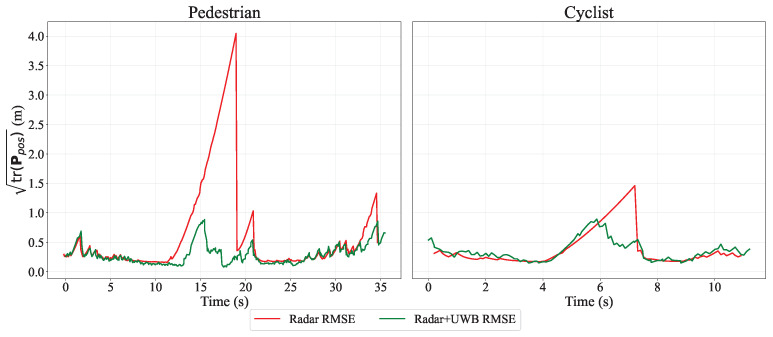
The square root of the covariance trace is plotted against time; lower values indicate greater filter confidence and lower positioning errors. Radar-only traces (red line) climb steeply during the absence of radar measurements due to the blind spot. Fusion traces (green line) remain steady below 1m during both cases, the cyclist and the pedestrian.

For the pedestrian run in Figure 6, the spike at the beginning is due to the EKF and UKF still adapting their parameters while missed radar measurements occur. In this phase, the filter output relies mainly on the UKF, where the motion model has not yet fully converged early in the track. The missing measurements can be confirmed through the elevated variance trace of the position in Figure 7 during these time steps.

In safety-critical applications, evaluating the statistical confidence of the localization estimate is as crucial as measuring the absolute tracking error. To quantify this internal certainty, we evaluated the square root of the spatial covariance trace for both the radar-only and sensor fusion configurations, providing an estimate of the positional uncertainty in meters. Specifically, the trace was computed by summing the variances of the planar Cartesian coordinates at each time step, formulated as follows:

(48) $$\begin{array}{c} P_{\mathrm{pos}}=\left[\begin{array}{cc} P_{11} & P_{13}\\ P_{31} & P_{33} \end{array}\right]=\left[\begin{array}{cc} \mathrm{Var}(x) & \mathrm{Cov}(x,y)\\ \mathrm{Cov}(y,x) & \mathrm{Var}(y) \end{array}\right], \end{array}$$

(49) $$\begin{array}{c} \mathrm{tr}\left(P_{\mathrm{pos}}\right)=\mathrm{Var}\left(x\right)+\mathrm{Var}\left(y\right)=P_{11}+P_{33}. \end{array}$$

where $P_{11}$ and $P_{33}$ denote the variances of *x* and *y* position states, respectively, in the covariance matrix P associated with the state vector x in (11). Figure 7 shows the position-uncertainty trace for the pedestrian and cyclist runs. In the radar-only configuration the covariance grows dramatically with time because, while the target is inside the radar’s blind-spot, the filter performs successive prediction steps without any measurement updates. By contrast, the fusion with UWB attenuation measurements constrains the uncertainty, keeping the square-root of the covariance trace below 1 m throughout the blind-spot region.

**Figure 8:**
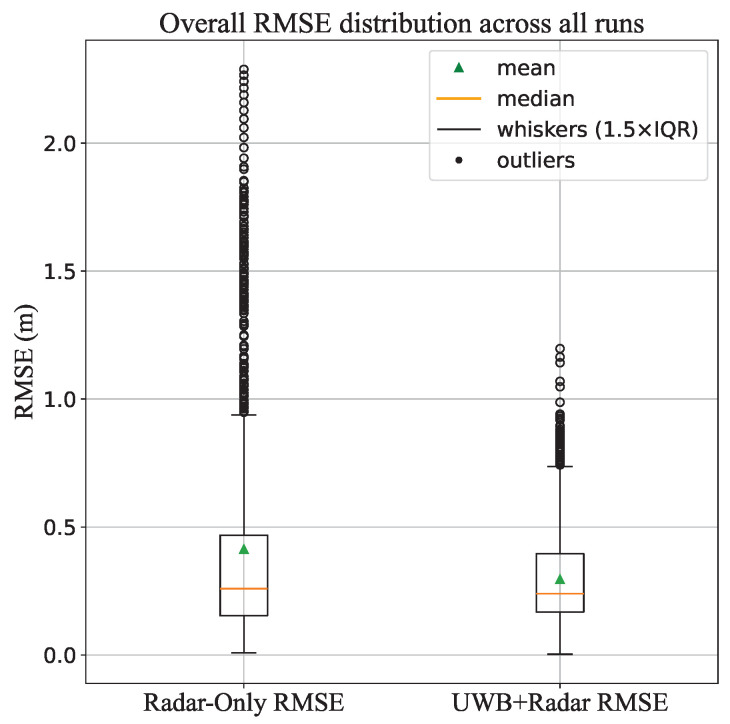
Box and Whisker plot shows the comparison between the distribution of the RMSE values of 7 runs, where the values of the fused data remains below 1m, except for few outliers. However, the distribution of the radar-only RMSE values exceeds the 1m threshold, as well as to outliers exceeding 2m.

The overall RMSE for the seven VRU runs for radar-only and radar–UWB fusion is shown as a Box and Whisker plot in Figure 8. It can be observed that the RMSE of the radar–UWB fusion remains below 1 m, with very few outliers exceeding the 1 m threshold by negligible values. In contrast, the RMSE for radar-only shows a higher overall RMSE, where not only its distribution exceeds the 1 m threshold, but its outliers lie even above 2 m.

**Figure 9:**
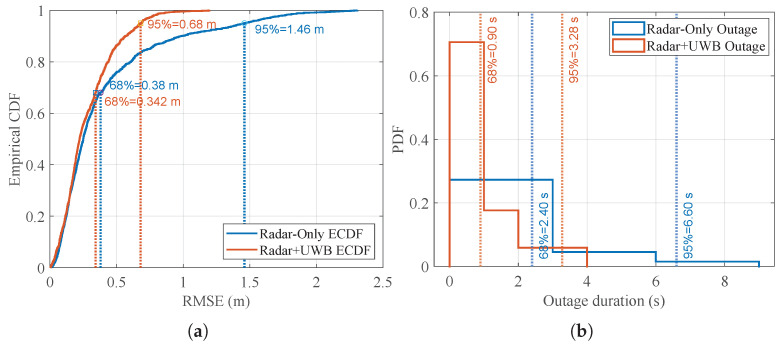
Performance comparison of radar-only and radar+UWB localization approaches in terms based on data collected from all runs. (**a**) illustrates the ECDF of RMSE showing percentile-based accuracy CEP68 and CEP95. And (**b**) illustrates the probability density function of the outage duration.

Figure 9 shows the empirical cumulative distribution function (ECDF) and the outage duration distribution of the RMSE for the two approaches. The ECDF in Figure 9a compares the RMSE distribution of radar-only localization with radar–UWB fusion approach. The ECDF curve shows the probability of the error being smaller than a specific error value (on the x-axis). We compared the two approaches in terms of the percentile-based accuracy metrics CEP68 (equivalent to 1σ) and CEP95. The results show that both approaches achieve similar accuracy around 0.34–0.38 m at 68%. However, radar–UWB fusion outperforms the radar-only approach at 95% achieving an RMSE below 0.68 m compared to 1.46 m. Hence, these experiments demonstrate that the fusion of radar and UWB data reduces the error tail and improves robustness.

Figure 9b shows the probability distribution of the outage duration. An outage is defined as the time interval within which the RMSE stays above a specific threshold and is given by the considered application. For a threshold of 0.4m, the outage at 68% with radar–UWB fusion is 0.9 s, while the radar-only yields a value of 2.4 s. At the 95th percentile, the outage is also reduced from 6.6 s to about 3.3 s. The results show that when the localization becomes unreliable, the radar–UWB fusion recovers faster while the radar-only approach suffers from longer error bursts.

## 5. Conclusions

This paper presented a novel radar and Ultra-Wideband (UWB) sensor fusion approach designed to enhance the safety of Vulnerable Road Users (VRUs) at intersections. By addressing the limitations of standalone sensors, this implementation serves as a highly reliable proof of concept for continuous VRU tracking in complex, mixed-traffic scenarios.

Managed by a hybrid Kalman Filter architecture, the proposed method significantly reduced VRU tracking uncertainty. While the Extended Kalman Filter (EKF) maintained real-time tracking on both the VRU and vehicles using radar data, the Unscented Kalman Filter (UKF) seamlessly integrated UWB measurements to bridge critical tracking gaps, most notably within radar blind spots. The experimental results demonstrate that fusing these modalities mitigates severe trajectory deviations, resulting in a precise and continuous detection and tracking performance.

Ultimately, these promising results validate the proposed fusion concept and establish a robust foundation for real-world traffic safety applications. By effectively resolving sensor-specific vulnerabilities, the system provides a redundant concept for robust road monitoring required to elevate VRU protection mechanisms and sets the basis for safer urban environments.

## Data Availability

The data presented in this study are available on request from the corresponding author due to ongoing developments and data-sharing restrictions.

## Abbreviations

The following abbreviations are used in this manuscript:

VRU	Vulnerable Road User
UWB	Ultra-Wideband
JCS	Joint Communication and Sensing
UKF	Unscented Kalman Filter
EKF	Extended Kalman Filter
V2VRU	Vehicle-to-VRU
LoS	Line-of-Sight
C-ITS	Cooperative Intelligent Transport Systems
CPMs	Cooperative Perception Messages
GDPR	General Data Protection Regulation
ADAS	Advanced Driver Assistance System
DFL	Device-Free Localization
MPCs	Multi-Path Components
MDFL	Multipath-Enhanced Device-Free Localization
RF	Radio Frequency
BMDV	Bundesministerium für Digitales und Verkehr (Federal Ministry for Digital and Transport)
CLS	Communication, Localization, and Surveillance
FMCW	Frequency Modulated Continuous Wave
FFT	Fast Fourier Transform
ADC	Analog/Digital Converter
RDM	Range-Doppler Map
CFAR	Constant False Alarm Rate
DBSCAN	Density-Based Spatial Clustering of Applications with Noise
RCS	Radar Cross-Section
PDA	Probabilistic Data Association
JPDA	Joint Probabilistic Data Association
TDMA	Time Division Multiple Access
CIR	Channel Impulse Response
RMSE	Root Mean Square Error
ECDF	Empirical Cumulative Distribution Function
